# Automated Non-Contact Respiratory Rate Monitoring of Neonates Based on Synchronous Evaluation of a 3D Time-of-Flight Camera and a Microwave Interferometric Radar Sensor

**DOI:** 10.3390/s21092959

**Published:** 2021-04-23

**Authors:** Johanna Gleichauf, Sven Herrmann, Lukas Hennemann, Hannes Krauss, Janina Nitschke, Philipp Renner, Christine Niebler, Alexander Koelpin

**Affiliations:** 1 Nuremberg Institute of Technology, 90489 Nürnberg, Germany; herrmannsv69694@th-nuernberg.de (S.H.); hennemannlu71902@th-nuernberg.de (L.H.); kraussha71215@th-nuernberg.de (H.K.); nitschkeja72965@th-nuernberg.de (J.N.); rennerph72935@th-nuernberg.de (P.R.); christine.niebler@th-nuernberg.de (C.N.); 2 Institute of High-Frequency Technology, Hamburg University of Technology, 21073 Hamburg, Germany; alexander.koelpin@tuhh.de

**Keywords:** non-contact monitoring, neonates, synchronous evaluation, respiratory rate

## Abstract

This paper introduces an automatic non-contact monitoring method based on the synchronous evaluation of a 3D time-of-flight (ToF) camera and a microwave interferometric radar sensor for measuring the respiratory rate of neonates. The current monitoring on the Neonatal Intensive Care Unit (NICU) has several issues which can cause pressure marks, skin irritations and eczema. To minimize these risks, a non-contact system made up of a 3D time-of-flight camera and a microwave interferometric radar sensor is presented. The 3D time-of-flight camera delivers 3D point clouds which can be used to calculate the change in distance of the moving chest and from it the respiratory rate. The disadvantage of the ToF camera is that the heartbeat cannot be determined. The microwave interferometric radar sensor determines the change in displacement caused by the respiration and is even capable of measuring the small superimposed movements due to the heartbeat. The radar sensor is very sensitive towards movement artifacts due to, e.g., the baby moving its arms. To allow a robust vital parameter detection the data of both sensors was evaluated synchronously. In this publication, we focus on the first step: determining the respiratory rate. After all processing steps, the respiratory rate determined by the radar sensor was compared to the value received from the 3D time-of-flight camera. The method was validated against our gold standard: a self-developed neonatal simulation system which can simulate different breathing patterns. In this paper, we show that we are the first to determine the respiratory rate by evaluating the data of an interferometric microwave radar sensor and a ToF camera synchronously. Our system delivers very precise breaths per minute (BPM) values within the norm range of 20–60 BPM with a maximum difference of 3 BPM (for the ToF camera itself at 30 BPM in *normal* mode). Especially in lower respiratory rate regions, i.e., 5 and 10 BPM, the synchronous evaluation is required to compensate the drawbacks of the ToF camera. In the norm range, the ToF camera performs slightly better than the radar sensor.

## 1. Introduction

### 1.1. Current Monitoring on the NICU

Preterm neonates on the Neonatal Intensive Care Unit (NICU) require continuous monitoring of their vital parameters such as heart and respiratory rate and the core temperature in order to allow an accurate assessment of their health status. When monitoring the respiratory rate, measurements are taken via electrocardiogram (ECG) electrodes (impedance pneumography), pulse oximeter, transcapnodes or even the ventilator itself. All of these methods involve direct contact with the baby’s skin and are cable-based. As preterm neonates are very underdeveloped and sensitive, those measurements can lead to risks such as pressure marks, eczema and skin irritations. Furthermore, the measurements can be imprecise if the baby sweats. The cable connection makes the handling of the baby more difficult, i.e., when weighing, changing the diapers and during cleaning. To increase the comfort of the baby, minimize the current risks and make the handling of the baby easier, a non-contact respiratory monitoring approach is developed.

### 1.2. State of the Art

Different approaches for monitoring the respiratory rate without contact using 2D imaging methods, optical depth measurements and radar are mentioned in the literature and are introduced.

#### 1.2.1. 2D Imaging Methods

There are several non-contact monitoring methods based on visual detection which have been tested on babies, such as thermal imaging [1,2,3,4,5] and Red Green Blue (RGB) cameras [6,7,8,9]. The main drawback of these methods is that the face needs to be visible and that RGB imaging is not independent of lighting conditions.

#### 1.2.2. Optical Depth Measurements

Optical depth measurements such as Red, Green, Blue-Depth (RGB-D) [10,11,12,13,14] and structured light plethysmography [15,16,17] have only been tested on adults and children older than two years. There is one publication by Hesse et al. which uses an RGB-D camera to track the body shape of moving infants [18]. The aim is to detect neurodevelopmental disorders such as cerebral palsy. This method could possibly be used for the respiratory rate detection if adapted.

Structured light and time-of-flight (ToF) cameras can be called 3D-cameras as they deliver 3D point clouds. The difference lies within the measurement principles. The structured light camera projects an infrared light pattern. The reflection is measured by the inbuilt infrared camera from a different perspective. The measured distortion can be used to calculate the depth points. A ToF camera sends out modulated light signals [19]. If an object lies in front of it, there will be a phase difference within the reflected signal which allows the calculation of the distance to that object. Both principles have the advantage that they are light-independent and do not require markers or the face to be visible as would be necessary for an RGB or thermal camera. Nonetheless, most of the proposed approaches using structured light [12,20] or ToF [21,22] have only been tested on adults. There are two approaches which use structured light cameras that have already been tested on babies. The first method requires two cameras and calculates the tidal volume [23,24]. In the next step, the respiratory rate is determined. Calculating the tidal volume is a complex approach and is not necessary if only the respiratory rate needs to be detected. For this, one camera would be enough. The other approach is only used with open incubators and requires the manual selection of measurement points to be used [25]. This means that the thorax is not automatically detected. This approach has only been tested on preterm neonates and is probably specialized only on the respiratory frequency range of neonates. Gleichauf et al. developed a non-contact respiratory monitoring approach based on structured light which is completely automatic and was tested and verified with a neonatal simulator [26]. Thus far, there is no approach for preterm neonates using a ToF camera.

It should be possible to carry out further developments with one of the optical depth measurements to allow an automated respiratory rate detection for neonates. Nonetheless, we are looking for a method which is also capable of detecting the heartbeat for an extended vital parameter monitoring system in the future.

#### 1.2.3. Radar

Another non-contact measurement method to detect the respiratory rate is radar. There are different types of radar signals: Continuous Wave (CW), Frequency Modulated Continuous Wave (FMCW) and Ultra-Wideband (UWB) [27]. CW continuously sends a high-frequency signal. FMCW is similar in the way that the signal is sent continuously, but its frequency is modulated periodically within a predefined frequency range. This allows the detection of distances. UWB sends short radar signal pulses or broadband sweep and receives the reflected signal in the interim. A special variant of CW radar sensors is the microwave interferometric radar sensor.

There are several approaches for measuring the respiratory rate which have been tested with toddlers and neonates using UWB radar. Kim et al. used an Impulse-Radio Ultra-Wideband (IR-UWB) radar device with a center frequency of 7.29 GHz and a bandwidth of 1.5 GHz for monitoring the respiration of six neonates [28]. Schleicher et al. tested their method based on IR-UWB with a frequency band between 3.1 and 10.6 GHz with male adults and one seven-week-old baby and showed that it is possible to record the breathing cycle [29]. In a patent by Tupin, an UWB monitor for neonates to detect respiratory and cardiopulmonary distress is presented [30]. The disadvantage of this method is that impedance converter pads have to be used to prevent the loss of UWB energy. The baby needs to be placed on top of these pads.

Several tests were made using FMCW radar for the vital sign detection with adults. Wang et al. used a FMCW radar with a center frequency of 80 GHz and bandwidth of 10 GHz with sawtooth modulation (which only allows measurement of distances) [31]. For the detection of the respiratory rate, the best results were achieved when the sensor was placed on the left side of the patient’s body. In the publication by Mathews et al., another non-contact monitoring system is presented which is based on a 35 GHz FMCW radar sensor [32]. The suggested areas of application include the military, the NICU and traumatic injuries and burn injuries, but it has not yet been tested on babies. The difficulty is the removal of artifacts caused by patient movements. One disadvantage using FMCW radar is that the patient has to stay still. This cannot be ensured for preterm neonates even though they generally do not move too much. Marnach et al. used a 24 GHz FMCW radar sensor to take measurements of a reflector placed on top of a speaker acting as simulator of a baby’s respiration [33,34].

Unmodulated CW radar can be used for the detection of vital signs, too. Li et al. developed a Doppler radar sensor (unmodulated, CW) with a frequency of 5.8 GHz which can also be used for monitoring babies and infants at home for preventing sudden infant death syndrome [35]. The application for preterm neonates is not explicitly mentioned. This system is used within an infant monitoring prototype. The radar sensor is positioned at the side of the baby’s bed. The remaining problem with this system is that, if the child moves and the detected movement due to respiration and heartbeat is superimposed by motion artifacts, it is difficult to receive correct and precise vital parameters. Thus, there is potential for improvement and further developments. In a next step, their system was verified using an infant simulator (high fidelity infant simulator METI, 3–6 months) [36]. In this case, the radar sensor was placed underneath the baby. Unmodulated CW radar is sensitive to considerable background noise caused by walking or talking subjects near the measurement environment. These movements can superimpose the vital signs and make the detection harder.

In a comparative study on UWB and CW radar for respiratory monitoring, it was shown that a CW radar module developed by the Sapienza—Università di Roma could detect a movement of 1 mm from a distance of 2 m. The used FMCW radar (IVS-148 of company InnoSent) could detect movements of 2 mm from a distance of 3 m. In comparison, the UWB module NVA-R640, which is used by the university of Oslo, could only detect a movement of 10 mm from a distance of 3 m [37]. This shows that CW radar can detect smaller movements from greater distances than UWB radar.

A so-called six-port-based CW radar sensor is even capable of measuring movement within the micrometer range [38]. The six ports include two input and four output ports and have the characteristic that two input signals are superimposed under four relative and static phase shifts which results in four sum signals as output [39]. From these phase shifts, the relative distance can be calculated.

Another reason for using CW radar based sensors for a monitoring system is that it allows the use of the Industrial, Scientific and Medical (ISM) frequency band which has been explicitly approved for scientific and medical applications. Working within the ISM band, a low-radiation power should be used. By law, a maximum radiation power of 100 mW is allowed.

In comparison, CW is easier to apply and allows an easier circuit technology than UWB but can lead to problems with multiple reflections when other moving objects are present in front of or behind the patient. This has to be removed by filtering and signal processing. Another advantage of a microwave interferometric radar sensor is that distance changes can be measured with micrometer precision by phase evaluation. Due to these advantages, a CW radar sensor based on microwave interferometric radar should be used. Similar radar sensors (six ports) have already been used for monitoring vital signs of adults, e.g., within the automotive sector [40]. They have also been tested within the clinical field and delivered good results [41].

#### 1.2.4. Laser Doppler Vibrometer

Marchionni et al. used a laser Doppler vibrometer to detect respiratory events of babies such as apnoea, irregular respiration and hiccups [42].

#### 1.2.5. Combination of Time-of-Flight and Radar

Nguyen et al. combined a Kinect2 (ToF) and a 2.4 GHz CW radar sensor based on the WARP kit v3 [43]. This WikiSpiro system was applied during sleep to determine the breathing volume of adults. The ToF camera did not calculate the breathing volume as it was only used to track the patient’s position, and these data were used to make sure that the radar sensor remained orthogonal to the patient’s thorax.

To sum up, we found that ToF cameras and microwave interferometric radar sensors based on CW show many advantages. ToF cameras do not require markers and are independent of lighting conditions. Microwave interferometric radar sensors can use the ISM frequency band and are also capable of measuring the heartbeat [44,45], which we want to do in the future. The radar sensor has the drawback that it is sensitive to motion artifacts due to, e.g., the baby moving its arms. If this movement has a similar frequency to the frequency of the thorax movement resulting from respiration, it can lead to issues. There are approaches for example by Tang et al. using a self- and mutually injection-locked (SMIL) radar system which allows random body movement cancellation, but this was only tested on adults [46]. Using SMIL radar requires a completely different hardware setup. As ToF cameras deliver 3D point clouds which allow tracking of the baby’s movement, the combination of a microwave interferometric radar sensor and a ToF camera could help to eliminate those motion artifacts. Furthermore, there is no system so far which combines and evaluates the data of a ToF camera and a microwave interferometric radar sensor synchronously for determining the respiratory rate and which has been tested on neonates.

#### 1.2.6. Neonatal Simulation System

In the following, existing neonatal simulation systems are presented, which can be used as a test system for non-contact respiratory rate monitoring.

The preterm infant simulator “Paul” was developed by SIMCharacters and is according to the manufacturer the most detailed simulation of a preterm neonate on the market at this point in time [47]. The simulator has the size of a 27 + 3-gestational-week-old preterm baby and is used for training doctors, nurses and midwives. Physiological parameters such as weight, size and inner organs are close to reality. One feature is the simulation of the respiration. This includes normal respiration between 0 and 100 BPM as well as pathological breathing such as caused by a pneumothorax, etc. The respiratory system of the simulator is made up of two test lungs which are connected to a ventilation system and can be parameterized. Furthermore, the coronary system can be parameterized and pathological conditions can be simulated. The pulse can only be measured using electrodes. This makes it impossible for a radar sensor to detect the heartbeat.

Similarly, “Premature Anne” by Leardal is a simulator system of the size of a 25-week-old premature neonate [48]. This simulator is also used for training. Unlike Simulator “Paul”, the respiration of the baby is not simulated. Only if mechanical ventilation takes place will the chest rise and fall.

The “3–6 months old infant simulator” by METI was used for testing a 5.8 GHz CW Doppler radar sensor [36]. The respiratory rate is simulated so the rise of the chest can be seen [49]. As the size and age of this simulator does not correspond to a newborn or preterm baby, this simulator does not fulfill our requirements.

The Norwegian company Novelda presents another approach with its “XeThru Bot” [50]. The robot is made up of LEGO^®^ MINDSTORMS^®^ components and is used for simulating the thorax movement of a baby. The motor moves a metal ball and is controlled by a LEGO^®^ MINDSTORMS^®^ microcontroller. For imitating the chest of the baby, a metal ball with a diameter of 2 cm is used. The ball is moved by a distance of 4 mm and its movement is evaluated by software. The “XeThru Bot” is capable of producing precise and reproducible movements. As in our case we needed a simulation system which can simulate the heartbeat as well as the respiration and both signals simultaneously, this concept is not sufficient for our requirements.

Marnach et al. and Schmiech et al. presented two simulator dummies used within the research project SINOPE-NEO [51]. As they were using radar sensors for the detection of vital signs, a speaker with a reflector on top was used with a fixed frequency of 50 BPM for the simulation of the respiratory rate [33]. The reflector allows a good radar reflection signal but does not represent the actual skin characteristics of a baby in terms of absorption. It is mentioned that a superimposition of two sine signals is possible, but the usage and application is not presented [34]. Additionally, a pneumatic doll with a balloon inside was used for first tests [33].

Within our research project NeoWatch (Funding reference: 13FH546IX6), we worked on a concept which simulates the movement of the thorax and the heartbeat using a hydraulic pump and a vibrating motor. A plastic container was covered by an elastic membrane and an air pump was connected. The membrane expands and contracts due to air being pumped into the container. This simulates the chest movement. On the membrane, a vibrating motor (piezo element) was attached to simulate the heartbeat. However, this concept was also not applicable as the vital parameters are not precise enough and could not be adjusted adequately.

Before testing on real babies, we needed to have a stable and robust sensor system for monitoring the respiratory rate. The functionality of the sensor system has to be verified by a neonatal simulator which is capable of simulating the respiration with different breathing patterns, the heartbeat and both signals at the same time. As the above-mentioned neonatal simulation systems do not fulfill our requirements or are too expensive, we decided to develop our own simulator. The development of our simulator is described in Section 2.

## 2. Materials and Methods

We now describe all components which are part of our ToF-radar system. First, the concept is presented, and then the hardware and software are described in more detail.

### 2.1. Concept and Theoretical Approach

In this section, the concept of our measurement system for non-contact respiratory monitoring is proposed. Our system is made up of a ToF camera, a microwave interferometric radar sensor and a neonatal simulator as gold standard. The measurement principles of a 3D time-of-flight camera and a microwave interferometric radar sensor are described. The theoretical concept of our neonatal simulator is presented.

#### 2.1.1. 3D Time-of-Flight Camera

There are two principles which can be used within time-of-flight (ToF) cameras: the impulse time-of-flight method and the phase difference method. The impulse time-of-flight principle calculates the distance *d* to the object from the time *t* of the impulse emitted until received:

(1) $$\begin{array}{c} d=\frac{t\;\cdot \;c}{2} \end{array}$$

with *c* as the speed of light.

The phase difference method is based on the phase shift caused by the reflected modulated signal. The distance to the object can be calculated using the phase shift ϕ and the wavelength λ of the modulated signal:

(2) $$\begin{array}{c} d=\frac{\phi }{2\;\cdot \;\pi }\;\cdot \;\frac{\lambda }{2} \end{array}$$

The principle used in our ToF camera is the phase difference method [52]. The modulated infrared light reflection is detected by the 3D imager. The 3D imager then measures the phase shift for calculating the distance to the target. The data result in a 3D point cloud. ToF point clouds are very sensitive to noise and can produce errors and artifacts caused by the characteristics of the camera or the surrounding area. Those errors can be grouped into systematic and non-systematic errors [53]. Non-systematic errors can be photocharge conversion noise, quantization noise or electronic shot noise [54]. Furthermore, multiple way reflection, i.e., at edges and light scattering, which can occur if a light object is positioned in front of a dark object, can lead to errors. Systematic errors such as circular distance errors, fixed pattern noise and amplitude related errors due to inhomogeneous lighting can be removed by calibration [53]. This means that filtering is needed to minimize the non-systematic errors and allow further point cloud processing. Typical filters used are the Median filter for noise reduction and a Statistical Outlier Removal filter [55] to remove outliers caused by reflections.

#### 2.1.2. Microwave Interferometric Radar Sensor

A special variant of CW radar sensors is the microwave interferometric radar sensor. Only the phase shift between the reference and the reflected microwave has to be measured and no Fourier transform is required. The relative distance can be calculated using the following formula:

(3) $$\begin{array}{c} \Delta x=\frac{\Delta \sigma }{2\;\cdot \;\pi }\;\cdot \;\frac{\lambda }{2} \end{array}$$

with Δx as relative distance, Δσ as relative phase shift and λ as wavelength.

#### 2.1.3. Neonatal Simulator

As mentioned above, our neonatal simulator needs to fulfill the following requirements:

- Size of preterm neonate: thorax area with a diameter of 70–100 mm
- Mean stroke of 2.0 mm for the respiration and a mean stroke of 0.13 mm for the heartbeat
- Frequencies between 0.083 and 4.17 Hz (5–250 BPM)
- Variable stroke size
- Displacement due to respiration and heartbeat
- Superimposition of both signals needs to be possible
- Different respiratory patterns such as *deep* and *normal* breathing

We considered two possible approaches: a mechanical approach (oval disk) and an electro-mechanical approach (speaker). Both concepts are presented in brief.

##### Mechanical Approach

The mechanical approach simulates the respiratory motion by moving a plate up and down using the rotation of oval disks underneath. The size of the oval disks has to vary so that different amplitudes can be simulated. The shaft is moved by a mounted motor on the right. The system control is provided by an Arduino microcontroller. The oval disks are fixed onto the shaft using clamping screws. The amplitude can be adjusted by moving the disks. The frequency of the simulated sine can be adapted by changing the speed of rotation. The coronary simulation is provided by a vibrating motor which is built into the plate.

##### Electro-Mechanical Approach

**Figure 1:**
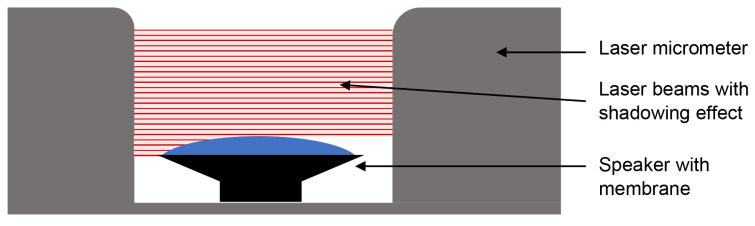
Electro-mechanical approach for the neonatal simulator. Whenever the membrane of the speaker moves and penetrates the light carpet the change can be detected.

**Figure 2:**
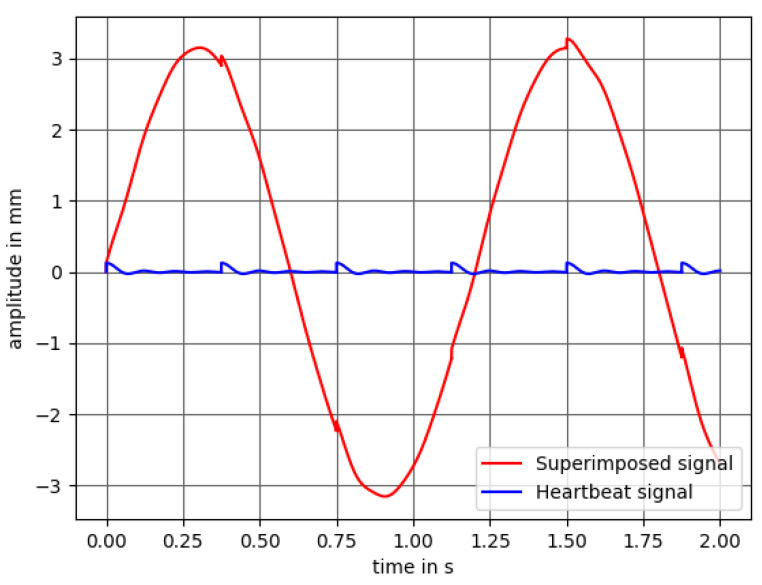
Heartbeat signal (**blue**) with a heart rate of 160 beats per minute and respiratory signal of 50 breaths per minute superimposed by the heartbeat signal (**red**).

This concept (see Figure 1) uses a speaker, an amplifier circuit, a computer and a laser micrometer. The respiration and heartbeat signals are generated by a software program on the computer. The idea is to use the superimposition of two sine waves—one for simulating the respiration and a decaying sine for the heartbeat. In Figure 2, the heartbeat signal (blue) and the respiratory signal superimposed by the heartbeat (red) can be seen.

It is assumed that the amplitude of the respiratory rate of a preterm neonate has a stroke of around 2 mm and the displacement caused by the heartbeat is around 0.13 mm. As there are no medical reference values available for the movement of the chest wall due to the heartbeat for neonates, we made an approximation. It is known that the chest wall displacement of adults ranges from 0.2–0.5 mm [56]. The dimensions of an adult heart are of around 12 cm × 8 cm × 6 cm [57]. A newborn heart has the size of a walnut with dimensions of around 3.5 cm × 2.5 cm × 2.5 cm [58]. As we are considering the chest wall movement in the z-direction, we assume that for a newborn baby it is multiplied by a factor of $\frac{2.5\mathrm{cm}}{6\mathrm{cm}}$. This leads to an approximated chest wall movement of 0.0834–0.2085 mm with a mean of 0.146 mm. We use a value of 0.13 mm as this is the smallest possible movement the speaker can provide lying within the approximated chest wall displacement range.

The superimposed signal can then be transferred from the AUX output of the computer to the speaker. Due to the small output power of the computer the signal needs to be amplified before being sent to the speaker. The speaker transforms the electrical signal into the displacement of the speaker membrane.

This approach has the advantage that different respiratory and heart rates can be simulated as well as the superimposition of the respiratory and heartbeat signal without having to make any mechanical changes. Additionally, different breathing patterns can be easily simulated. For these reasons, we decided to implement the electro-mechanical approach.

To allow our neonatal simulator to be used as gold standard, a precise reference measurement has to be included. This reference sensor needs to be capable of detecting small movements within the submillimeter range so that thorax movements due to the heartbeat can also be detected. An excellent choice is a laser micrometer which uses the principle of shadowing [59,60]. Whenever an object penetrates the light carpet, the change can be detected. The laser micrometer is ideal for our test setup but cannot be used as a measurement device within the incubator on the NICU as it has a laser class 1 M, which means that the laser becomes dangerous if optical instruments magnify the laser. In addition, the baby would have to lie on the metal bar connecting the receiver and transmitter of the laser micrometer, which would be uncomfortable. Furthermore, erroneous measurements would occur if the baby sleeps on its side. For our neonatal simulator under ideal conditions, these points are negligible.

#### 2.1.4. Synchronous Evaluation for the Detection of the Respiratory Rate

The idea is to combine and evaluate the calculated BPM values from the radar sensor and the ToF camera synchronously and compare them with the gold standard. The distance signals over time will be compared to see how precise each measurement is. We expect that the combination of the radar sensor and the ToF camera increases the robustness of the respiratory rate detection and that the system will be more stable as the drawbacks of each sensor can be compensated for by the other.

### 2.2. Hardware Setup

**Figure 3:**
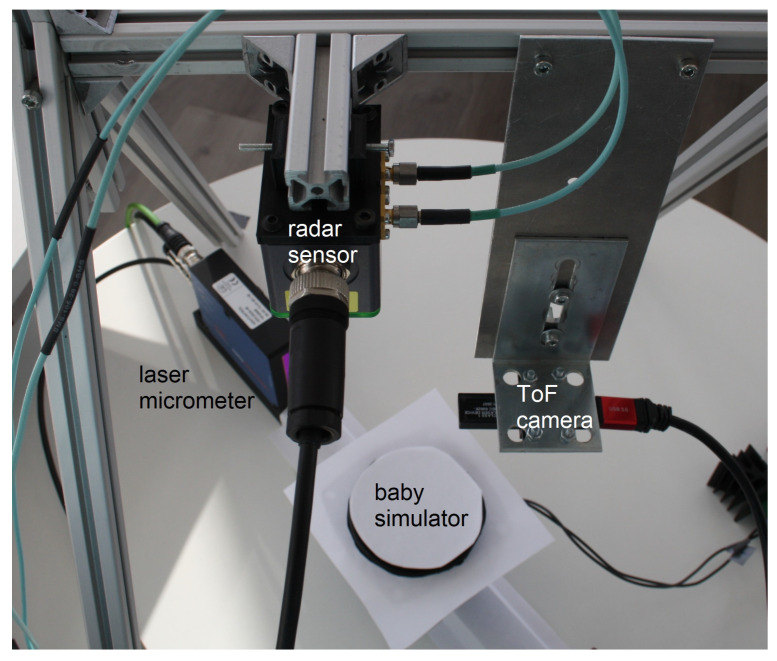
Hardware setup for the ToF-radar system. The baby simulator can be seen in the middle, and the laser micrometer serves as reference measurement.

Figure 3 shows the complete hardware setup made up of the radar sensor, the ToF camera and the baby simulator. The baby simulator is placed between the emitter and receiver of the laser micrometer. As can be seen, it is covered with paper in order to reduce reflections within the ToF point cloud arising from any dark object in front of a light surface. The radar sensor is positioned at a distance of 36.5 cm from the table and the ToF camera at a distance of 23.5 cm. Those dimensions correspond to the distances within our incubator. All different sensors are now described in detail.

#### 2.2.1. 3D Time-of-Flight Camera

**Figure 4:**
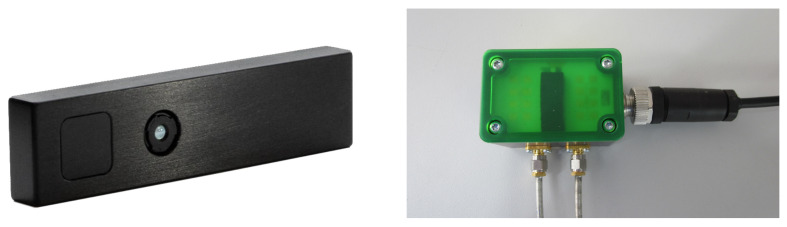
Pico flexx 3D time-of-flight camera by pmd [62] (**left**) and radar sensor by InnoSenT (**right**).

The 3D time-of-flight (ToF) camera used is the CamBoard pico flexx by the company pmd (see Figure 4). The camera has a measurement range of 0.1–4 m, a field of view (FoV) of 62° × 45° and a resolution of 224 × 171 (38 k) pixels. A frame rate of up to 45 fps (3D frames) is given. The camera is connected via USB to an Ubuntu 16.04 computer with Robot Operating System (ROS) Kinetic installed. The used driver is the Royale SDK provided by the manufacturer as well as the ROS package *pico_flexx_driver* [61]. For our purpose, a frame rate of 35 fps is set.

#### 2.2.2. Microwave Interferometric Radar Sensor

The microwave interferometric radar sensor used is the iSYS-4001 sensor by the company InnoSenT (see Figure 4). This sensor was specifically manufactured for the research project GUARDIAN [63], which uses radar for the monitoring of vital signs of patients in the palliative care unit. The radar sensor has a frequency of 24.2 GHz and delivers a power of 100 mW (20 dBm) which lies within the legally approved norm range. The signals from the radar sensor are received and transferred using an Infineon XMC4500 microcontroller and digitized by an analog-to-digital converter (ADC), ADS1298 by Texas Instruments, as it has a better performance than the inbuilt ADC of the Infineon XMC4500 microcontroller. This is done with a sampling rate of 2 kHz. The digitized values are then sent via Ethernet.

For integrating the radar sensor into the measurement system, a new ROS node was implemented. It provides all necessary functions for communication with the radar sensor via User Datagram Protocol (UDP). The sensor has two main modes of operation, sampling for an infinite and predetermined time. Before sampling, the ADC gain and the sensor’s sampling rate can be set. After every 50 samples, the sensor sends a new UDP message, containing four pairs of 24 Bit in-phase (I) and quadrature (Q) signals with 50 samples each. These are then converted to the receiving machine’s specific 32-Bit integer representation. For every UDP message, a new ROS message is then published containing the respective 50 samples.

#### 2.2.3. Neonatal Simulator

**Figure 5:**
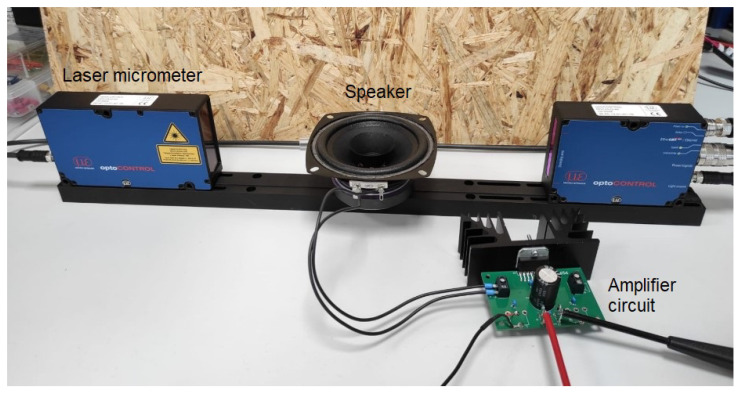
Neonatal simulator made up of an amplifier circuit with cooling body, a speaker and the laser micrometer.

The neonatal simulator is made up of several components (see Figure 5): a computer with an Ubuntu 16.04 operating system with the software to control the simulator installed, an amplifier circuit, a speaker and a laser micrometer. All components are now described in detail.

The amplifier circuit used has an output power of 2 × 35 W and a maximum current of 7.5 A [64]. A cooling body is used so that the produced heat will not interfere with the amplifier circuit.

The speaker used is by Visaton and has a frequency range of 80–20,000 Hz [65]. This low frequency range is important so that slow movements such as the respiration can be simulated. The speaker has a diameter of 10.16 cm, which corresponds to the size of a preterm neonate’s chest. On top of the speaker membrane, a construction made up of a table tennis ball cut in half and a circular cardboard cutout is positioned. The cardboard is covered with a disposable glove which has similar absorption characteristics to the skin of a baby.

The neonatal simulator is controlled by a Python script running on the computer which sends the respiratory and/or heartbeat signal. It can be either started from a graphical user interface (GUI) or via the terminal. The heart and respiratory rate to be simulated can be chosen from a drop down menu. Furthermore, *deep* respiration can be simulated which has a stroke of around twice the magnitude of the *normal* respiration mode. Additionally, the heart and respiratory signals can be superimposed by adding the two sine signals.

The laser micrometer used for reference measurement is the optoCONTROL 2520 by MICRO-EPSILON [66]. It has a measurement range of 46 mm and a maximum resolution of 1 μm. The laser is of laser class 1M, which means that there is no harm to the user if the laser beams are not magnified by any optical instrument. The receiver and transmitter of the laser micrometer are connected via a CE2520-1 cable. Furthermore, a 24 V power supply is needed. The optoCONTROL is connected to a Microsoft Windows 10 laptop via Ethernet. MICRO-EPSILON provides the measurement tool ODC2520 DAQ tool, which was installed on the laptop. The tool automatically searches for a laser micrometer and connects to it. The measurement program needs to be configured via a web browser by simply entering the IP. For measuring movement with the sensor, the mode “Edge light-dark” needs to be selected. Once all settings are made, the ODC2520 tool can be started.

### 2.3. Software Algorithms

This section describes the developed signal processing software to determine the respiratory rate using a ToF camera and a radar sensor. The time synchronization with the gold standard reference is described.

#### 2.3.1. Time-of-Flight Camera for the Detection of the Respiratory Rate

In previous work, we described a method which determines the respiratory rate of neonates using a structured light camera [26]. As this camera was not suitable for the distances present within an incubator, the pico flexx time-of-flight camera by pmd was introduced which is capable of detecting objects up to 10 cm close. Both cameras deliver a point cloud which can be processed in order to calculate the respiratory rate.

As the ToF camera has different properties and a higher sensitivity towards noise and reflections, the original algorithm had to be extended by further filtering. Another difference to the structured light setup is that the dimensions of our neonatal simulator are smaller than the SimBaby by Laerdal which was used before.

**Figure 6:**
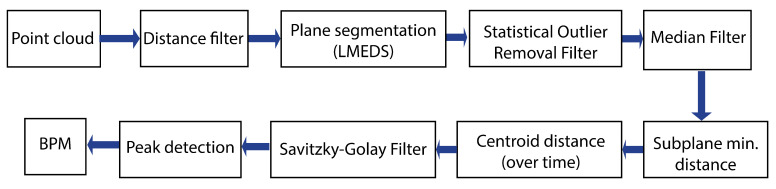
Signal processing for the respiratory rate detection using the ToF camera.

**Figure 7:**
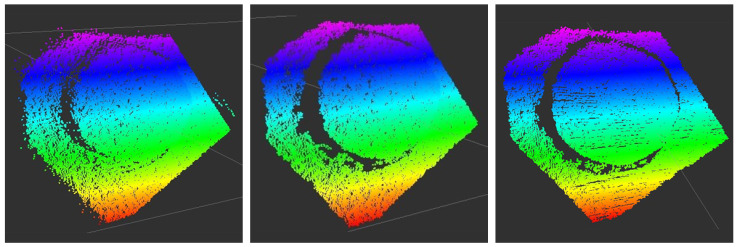
Plane without filtering (**left**) and with applied Statistical Outlier Removal Filter (**middle**). It can be seen that outliers at the edges are removed. When applying the Median filter, further smoothing of the point cloud occurs (**right**).

The signal processing flow chart for the respiratory rate detection using the ToF camera can be seen in Figure 6. The ToF camera delivers a 3D point cloud. First, a *distance filter* is applied to remove all points which are further than 20 cm away from the camera as these points would correspond to the table plane and are not of interest. In the next step, a *plane segmentation* from the Point Cloud Library (PCL) based on the Least Median of Squares (LMEDS) is applied [67,68]. The chosen model type is called *SACMODEL_PLANE*. The distance threshold is set to 2 cm and specifies that points with a maximal distance of 2 cm from each other belong to the same plane. To remove outliers caused by reflections at the edges, a *Statistical Outlier Removal filter* [69] with a standard deviation multiplier of α=1 and k=30 neighbors is used. For noise reduction, a *Median filter* [70] is applied with a *maximum_allowed_movement* of 3 mm (distance that a depth pixel is allowed to move when the filter is applied). In Figure 7, the effect of the Statistical Outlier Removal filter and the Median filter can be seen.

The distance point with the minimal distance to the camera is determined and a subplane with all points lying 4 mm away (into the z-direction) from the minimal distance is calculated. The centroid of this subplane is then plotted over time. This is the displacement caused by the respiratory movement. Packages of 33 centroid values over time are collected. For the first three packages, the number of breaths per minute (BPM) is determined using a *peak detection* [71] (taken after the synchronization signal). The respiratory rate (RR) in BPM is calculated by dividing the number of peaks by the time in seconds and multiplying by 60 s:

(4) $$\begin{array}{c} RR=\frac{breaths}{time}\;\cdot \;60 \end{array}$$

**Figure 8:**
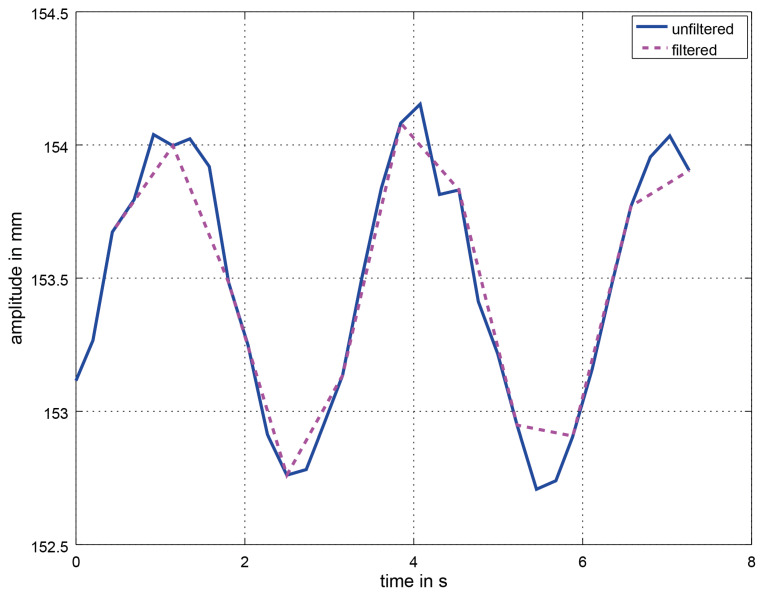
Unfiltered (**blue**) and filtered (**magenta**) section of the respiratory signal at 20 BPM in *deep* mode. The Savitzky–Golay filter has a window size 3 and polynomial order 2.

The mean of the BPM values of the three packages is used to parameterize the *Savitzky–Golay filter* based on Gram polynomials [72,73] in order to remove artifacts. Depending on the first BPM value, the window size and polynomial order varies (see Table 1). The grade of filtering depends on the frequency as there is more noise within the lower frequency region which means that a greater measure of filtering is needed. The ideal filter parameters were determined empirically. In Figure 8, a section of the unfiltered and the filtered respiration signal at 20 BPM in *deep* mode is displayed. From then on as before, a *peak detection* is used for the filtered values to determine the number of peaks within one data package over time. Once there are 18 packages (of 33 values each) with the Savitzky–Golay filter applied, a moving average window is used which moves by one data package of 33 values.

#### 2.3.2. Radar Sensor for the Detection of the Respiratory Rate

**Figure 9:**
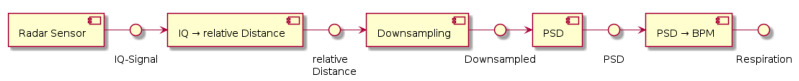
Signal processing for the respiratory rate detection using the radar sensor based on the Power Spectral Density (PSD) calculated from FFT.

In Figure 9, the signal processing for the respiratory rate detection using the radar sensor is visualized. The radar sensor delivers an in-phase and quadrature (IQ) signal which can be displayed in the following form:

(5) $$\begin{array}{c} I=r\;\cdot \;\cos(\sigma )=ℜ(Z) \end{array}$$

(6) $$\begin{array}{c} Q=r\;\cdot \;\sin(\sigma )=ℑ(Z) \end{array}$$

Using the in-phase and quadrature method for demodulation, the relative phase shift Δσ of the two signals can be calculated:

(7) $$\begin{array}{c} \Delta \sigma =\arctan\left(\frac{ℜ(Z)}{ℑ(Z)}\right)=\arctan\left(\frac{I}{Q}\right) \end{array}$$

In the first step, the relative distance is determined as follows:

(8) $$\begin{array}{c} \Delta x=\frac{\Delta \sigma }{2\;\cdot \;\pi }\;\cdot \;\frac{\lambda }{2} \end{array}$$

with Δx as relative distance, Δσ as relative phase shift and λ as wavelength.

The distance values are filtered using a low-pass filter with a cut-off frequency of 7 Hz which is then down sampled to 20 Hz. Then, a Fast Fourier Transform (FFT) is used to receive the frequency spectrum of the signal. The implementation is based on the fftw3 library [74]. The FFT uses the last 5 s which corresponds to 100 values at 20 Hz. Zero padding onto 2000 values takes place which means that the FFT is calculated with 2000 values with 1900 being set to zero. This allows a spectral resolution of 0.01 Hz. The FFT algorithm delivers 1000 complex values of which the Power Spectral Density (PSD) is calculated (${\left(ℜ\right)}^{2}+{\left(ℑ\right)}^{2}$). To receive the frequency with the highest peak, a peak detection based on a quadratic Savitzky–Golay [72] with window size 1 is applied. This delivers the first and second derivatives, which can be used to find out if a local maximum has been reached. The highest peak within the spectrum equals the respiratory frequency in Hz. All peaks below 10% of the highest peak and below 0.08 Hz are ignored. The last step is the conversion from Hz to BPM.

#### 2.3.3. Time Synchronization of the Different Sensors

The ToF camera and the radar sensor are started by the same launch file and automatically get ROS timestamps (Unix time). The camera and radar sensor, the neonatal simulator and the ROS bagfile recording is started using a bash script. Furthermore, a synchronization signal is included in the form of a sine of 180 BPM that lasts for 1 s at the beginning before the respiratory simulation starts. This signal is needed for synchronization with the laser micrometer. Between the synchronization signal and the actual measurement, a short fixed time sleep phase ensures that the synchronization does not influence the measurement data.

The measurements taken by the laser micrometer (time interval from start time and distance in mm) sampled at a rate of 2500 can be saved as a csv-file. For synchronizing the laser micrometer with the other sensors, the start time of the measurement is first converted to Unix time using a bash script and all timestamps are calculated by adding the time interval from the csv-file. The data are read by a ROS node which converts the csv-file to a ROS topic that can then be published together with the other data.

When calculating the distance signal from the ToF camera and the radar sensor, each distance point gets the correct bagfile timestamp set. It is then possible to display the synchronized laser micrometer, ToF and radar distance signal within *rqt_plot*.

### 2.4. Measurement Series

We recorded radar, ToF camera and laser micrometer data with our neonatal simulator. Different modes were used, *deep* and *normal* mode, with the stroke size in *deep* mode being approximately twice as large as in *normal* mode. Recordings of 15 min were taken of the following respiratory rates: 5, 10, 20, 30, 40, 45, 50, 55, 60 and 80 BPM. The norm range for respiratory rates in infants and neonates is between 20 and 60 BPM. The values above and below were tested to make sure the system also works in those ranges. To make sure that the reference laser micrometer delivers correct values, the number of peaks within a minute were counted manually. This showed that the set respiratory rate of the simulator corresponded accurately to the measured reference value. Furthermore the stroke size at each respiratory frequency was measured with the laser micrometer. The results can be found in Table 2. As can be seen, the stroke size is dependent on the set frequency. This behavior occurs as the stroke is proportional to the set voltage.

## 3. Results

In this section, the results from our ToF-radar system for detecting the respiratory rate is presented. Firstly, our algorithms were tested with all recorded bagfiles, and the BPM determined with the ToF camera approach and the radar approach were compared. In the second step, the distance signals of all measurement types were evaluated synchronously.

### 3.1. BPM Comparison

A new respiratory rate value is required at least every 5 s (this is what current NICU equipment delivers). The ideal is a new value every 3 s. The radar algorithm delivers a new BPM value every 50 ms. The ToF camera algorithm calculates a new BPM value around every 7.5 s. This value is low compared to our original approach with the structured light camera where we received a new value every 3 s [26]. This can be explained by the extension with the Median and Statistical Outlier Removal filters which take up more processing time. Furthermore, we now calculate a new subplane, which we did not before. As we are talking about the synchronous evaluation of radar and ToF in combination, we receive new values at the required rate.

**Figure 10:**
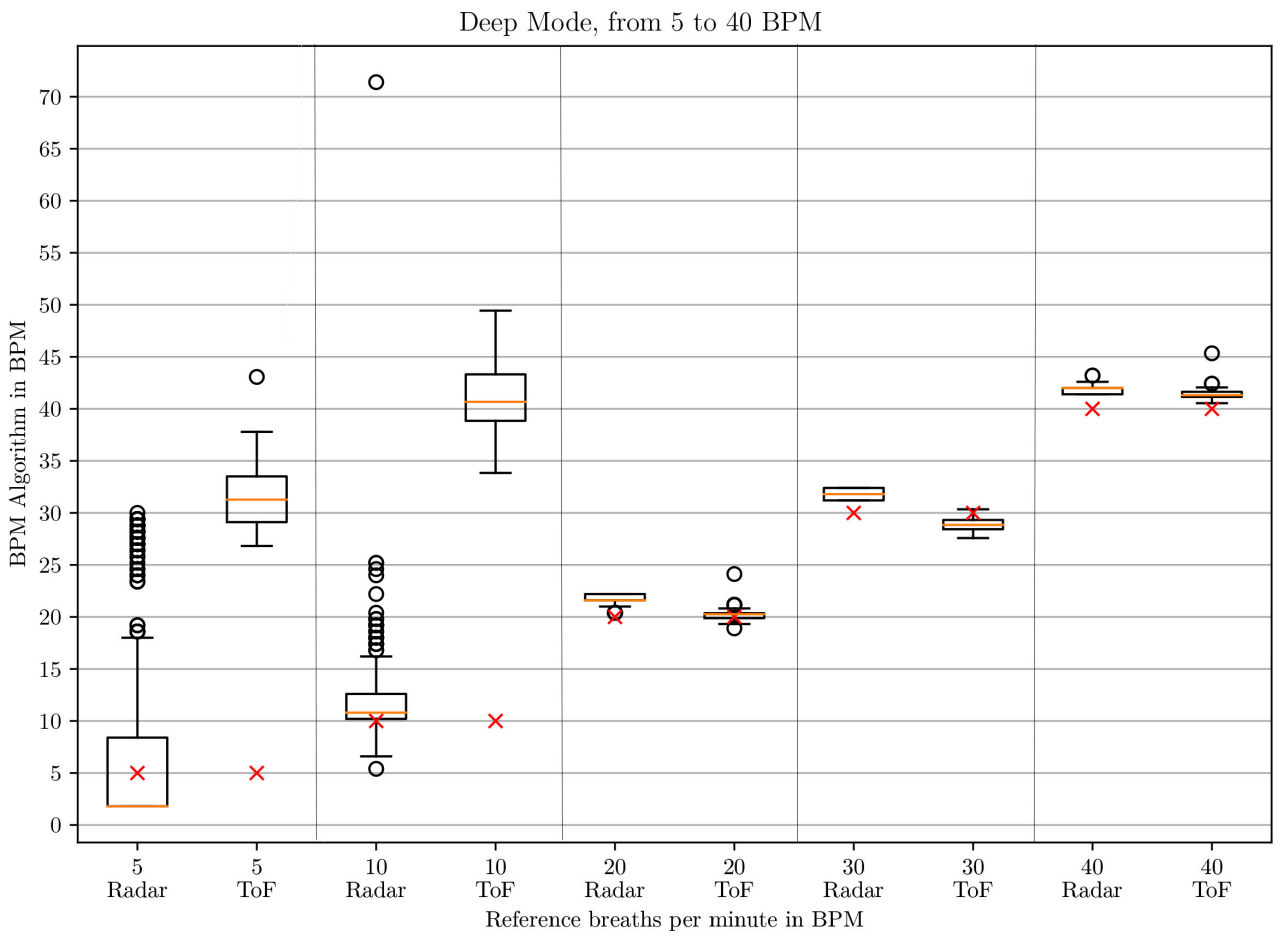
Box plot comparing the BPM values of the ToF and the radar algorithm for respiratory rates between 5 and 40 BPM in *deep* mode.

**Figure 11:**
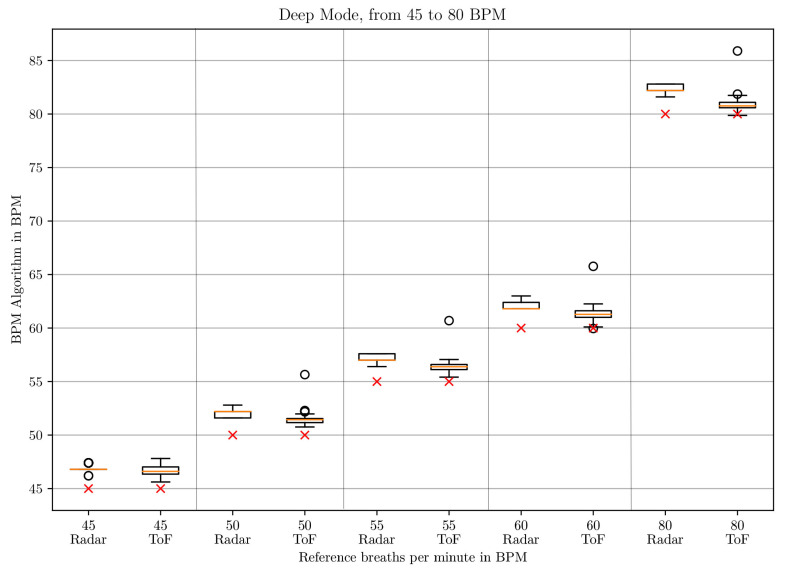
Box plot comparing the BPM values of the ToF and the radar algorithm for respiratory rates between 45 and 80 BPM in *deep* mode.

**Figure 12:**
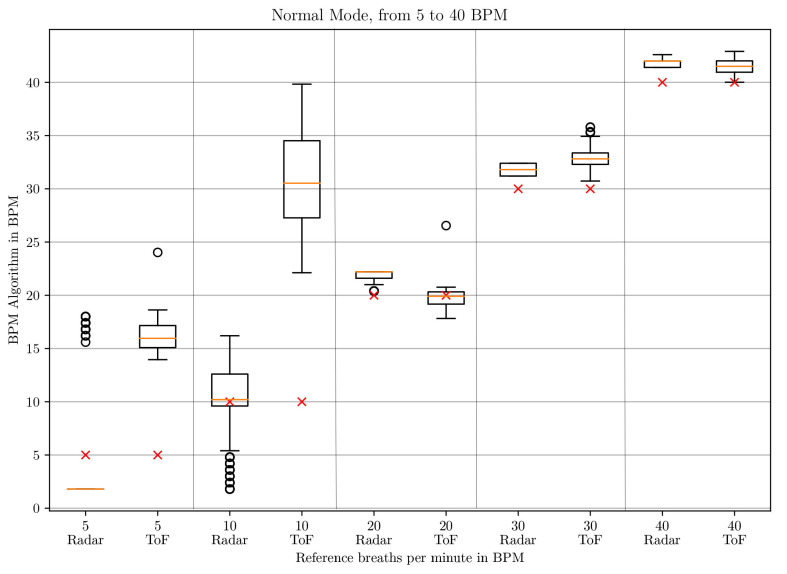
Box plot comparing the BPM values of the ToF and the radar algorithm for respiratory rates between 5 and 40 BPM in *normal* mode.

**Figure 13:**
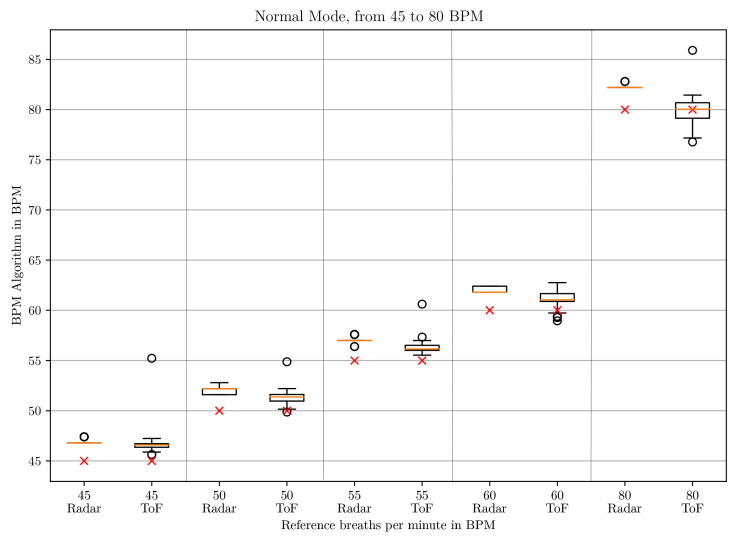
Box plot comparing the BPM values of the ToF and the radar algorithm for respiratory rates between 45 and 80 BPM in *normal* mode.

The box-whisker plots of the BPM values in *deep* mode in Figure 10 and Figure 11 and in *normal* mode in Figure 12 and Figure 13 display the median, minimum, maximum, upper and lower quartiles and outliers (marked as circles). The red crosses mark the position of the reference BPM. We compare the values from the ToF and the radar algorithm.

As can be seen for low respiratory rates (5 and 10 BPM), the ToF camera shows large differences to the reference BPM. In *deep* mode, there is a difference of 27 and 31 BPM to the set rates, respectively. In *normal* mode, there are differences of 11 and 21 BPM, respectively. The result depends on the first BPM value which determines the parameterization of the Savitzky–Golay filter. For 5 and 10 BPM in *deep* and 10 in *normal* mode, the starting values are larger than 32 BPM, which means that no filter is applied at all. For 5 BPM in *normal* mode, the starting BPM value lies at 24.0245 BPM, which means that the Savitzky–Golay filter with window size 3 and polynomial order 2 applies. This represents a stronger filter. Due to the strong noise of the distance signal at lower frequencies, the peak detection registers too many peaks. Another aspect influencing the start BPM value is the time over which the peak detection is applied. When testing on two different computers, we realized that the times for the same amount of data points differed. This is due to the ROS processes which run in the background and can lead to time variations. Unfortunately, ROS does not work in real-time. Even when working just on one computer, the time taken to process one batch of 33 data points can differ slightly.

Between 20 and 80 BPM, the results with the ToF approach are slightly better than the radar results. For small frequencies, radar performs better and shows a maximum difference of 3 BPM compared to the reference but with great outliers. The comparison is made to the median values.

### 3.2. Distance Signal Comparison

**Figure 14:**
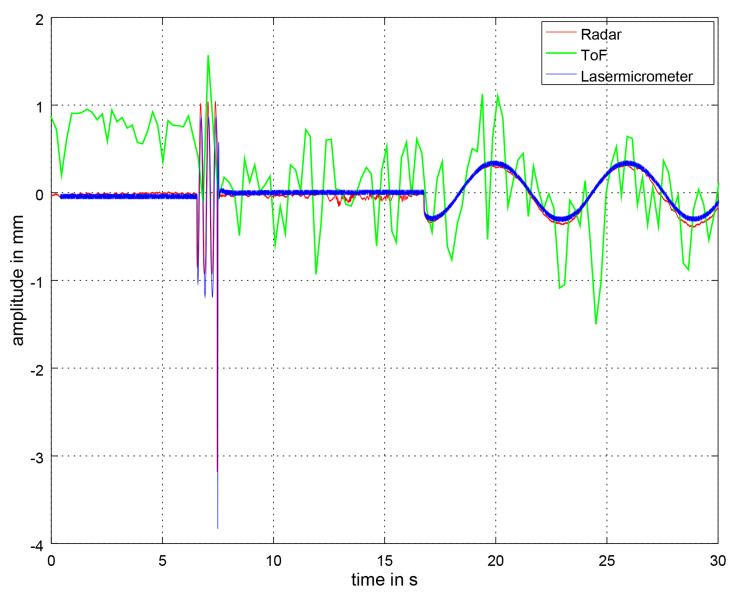
Unfiltered section of the respiratory signal at 10 BPM in *deep* mode measured by the ToF camera, the radar sensor and the laser micrometer. In the context of the ToF camera, unfiltered means that the Savitzky–Golay filter has not been applied yet. At the beginning, the synchronization signal can be seen. The ToF camera signal is very noisy and has high amplitude jumps. In comparison, the radar and laser micrometer signals are very close.

In this section, we compare the distance signals generated by the laser micrometer, the ToF and radar algorithms. Figure 14 displays a section of the respiratory signal at 10 BPM in *deep* mode. At the beginning, the synchronization signal is visible. The signals are unfiltered which means in the context of the ToF camera that the Savitzky–Golay filter has not been applied yet. This first section after the synchronization signal is used for determining the BPM for the parameterization of the Savitzky–Golay filter based on a peak detection. As the signal is very noisy, the detected BPM value will be very high (see BPM box plots). The radar signal is also slightly noisy, but, with the FFT approach, it is easier to detect the underlying main frequency. To receive the plotted radar amplitude, an Ellipse fitting was applied to the data twice afterwards. Ellipse fitting means that the IQ-values from the radar sensor are fitted onto an ellipse in order to calculate the transformation to the IQ unit circle. It serves as calibration. Our implementation is based on a MATLAB implementation by Gal [75].

**Figure 15:**
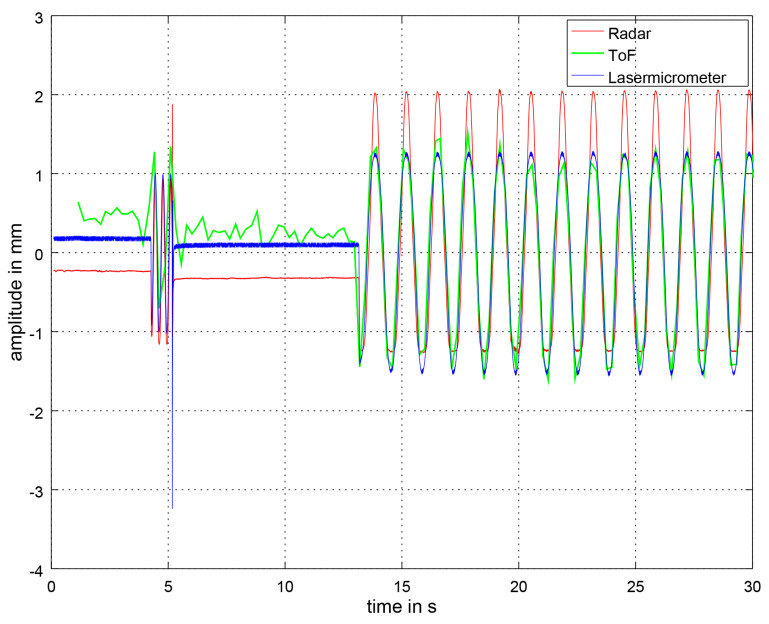
Unfiltered section of the respiratory signal at 45 BPM *deep* measured by the ToF camera, the radar sensor and the laser micrometer. In the context of the ToF camera, unfiltered means that the Savitzky–Golay filter has not been applied yet. At the beginning, the synchronization signal can be seen. At the beginning of the ToF signal, the noise is clearly visible. Later on, the stroke of the simulator thorax is higher compared with the noise. The amplitude is very similar to the laser micrometer. The amplitude of the radar sensor is slightly shifted and uneven. This might be due to the effects of the applied Ellipse fitting.

In Figure 15, the respiratory signal at 45 BPM in *deep* mode is plotted. The ToF signal looks very similar to the laser micrometer signal and is in phase. The radar amplitude seems to be asymmetrical but this does not have any effect on the FFT and the calculation of the BPM. In this case Ellipse fitting was also applied to the data afterwards. We found that the size of the radar amplitude depends on whether Ellipse fitting had been applied or not.

**Figure 16:**
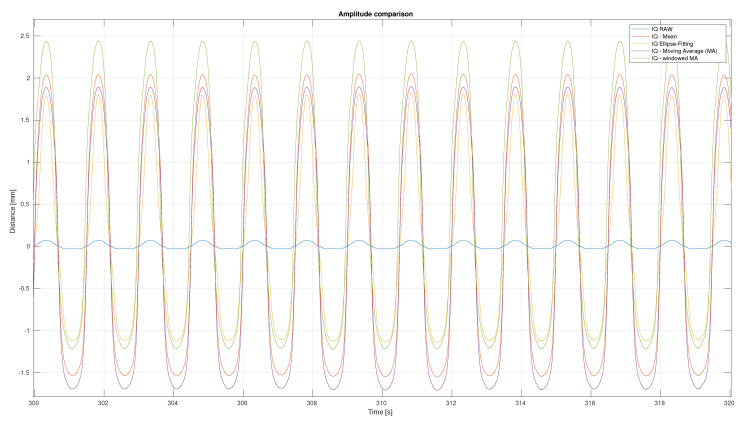
Radar amplitude variation depending on whether Ellipse fitting, the mean, moving average or the windowed average is used. Here, a respiratory rate of 40 BPM *deep* is used.

We tested at 40 BPM *deep* showing the dependency of the amplitude size with respect to Ellipse fitting, an applied mean, moving average and windowed average. All of these measures were applied to the IQ-data of the radar sensor. The results can be seen in Figure 16. We wanted to receive the highest amplitude possible. As the moving average and the windowed average had negative effects when applied to lower respiratory rates and the FFT delivered worse BPM values, Ellipse fitting was applied. Thus far, it is not possible to generate a stable and well fitting ellipse in real-time. This meant that an ellipse was calculated out of the whole radar data of one bagfile, and this ellipse was applied to the data afterwards.

## 4. Discussion

In this section, we discuss our results and compare them to our theoretical approach and the requirements we had. A comparison to the state of the art is also given.

### 4.1. Comparison with the Theoretical Approach

Even though several drawbacks of our sensors were known in theory, these effects had a greater impact in practice than expected. This led to several unexpected limits of our algorithms, which are now described.

We did not expect that the ToF camera would be as sensitive to noise and reflections compared with the structured light camera we used in previous work. Even though measures were taken such as covering dark surfaces of the simulator with paper to reduce reflection due to high contrasts and additional filtering, the distance signal for small respiratory rates is very noisy. For low respiratory rates, the stroke of the simulator is very small (as the stroke is proportional to the set voltage applied to the speaker). This means that the signal-to-noise ratio (SNR) is low. The peak detection applied cannot handle these signals well and delivers inappropriately high values which cause an incorrect parameterization of the Savitzky–Golay filter. As the Savitzky–Golay filter has fixed limits which also need to work for higher frequencies, the limits cannot be further shifted because then the filtering for higher rates would be too strong. It would have been expected that the results for small respiratory rates were better in *deep* mode than in *normal* mode due to a greater stroke.

We also did not expect that the publishing rate of a new BPM value of the ToF camera would only deliver a new value around every 7.5 s and that the internal ROS timing would have an effect on the calculation of the BPM value.

For radar, we did not expect that the application of the Ellipse fitting would influence the amplitude of the distance signal to this extent. It was also unexpected that the ToF sensor would perform slightly better within the norm BPM range.

We showed that each sensor has its strengths and weaknesses which complement each other well when determining the estimates of the BPM. Our results are a good basis on which can be built to receive a greater robustness by combining both sensors.

We require a new BPM value at least every 5 s. Our system delivers a new value every 50 ms which is sufficient. We realized that, when the set respiratory rate is very low, the calculated BPM values with the ToF algorithm are too high. However, the lower value of both sensors must be taken into account as these values may represent deranged physiological responses that may indicate a risk to the life of the baby, who should be checked by a doctor.

### 4.2. Comparison with the State of the Art

With this publication, we prove that we are the first to evaluate the data of a microwave interferometric radar sensor and a ToF camera synchronously for determining the respiratory rate. As mentioned in the state of the art, WikiSpiro does not evaluate the data of both sensors synchronously to determine the respiratory rate [43]. The ToF camera is only used for tracking the position of the patient to allow proper positioning of the radar sensor. Furthermore, their system has only been applied to and tested in adults.

Our system delivers very precise BPM values within the norm range of 20–60 BPM with a maximal difference of 3 BPM for the ToF camera at 30 BPM *normal*. In addition, we showed that ToF performs slightly better in the norm range than the radar sensor.

## 5. Conclusions

We showed that it is possible to evaluate the data of a radar sensor and a ToF camera synchronously and that the respiratory rate detection is very precise compared to our gold standard. We also showed that both sensors complement each other well and that a greater robustness can be expected when the drawbacks of each method are compensated for. Radar is better for low respiratory rates than ToF and also delivers a higher publishing rate of the BPM values. However, there are known issues in the literature when the baby moves (motion artifacts). Radar is also capable of detecting heartbeat, which we would like to address in the future. A further improvement for the radar algorithm would be a real time Ellipse fitting for the radar sensor. ToF delivers slightly better BPM values within the norm range than the radar sensor, but it has issues with low respiratory rates. This could be improved by also using a FFT instead of a peak detection, which would also mean that the ROS time variation could be neglected. The ToF camera additionally delivers a full 3D point cloud which can be used to reconstruct the baby’s position. Tracking the movement could be used to reduce motion artifacts to allow more robust radar sensor data.

It is planned to test our approach in the future on real babies within the NICU and to see how fast our system responds to changing respiratory rates.

## Figures and Tables

**Table 1:** Parameters for Savitzky–Golay filter.

BPM	Window Size	Polynomial Order
≤19	11	10
19–32	3	2
>32	None	None

**Table 2:** Measured strokes with the laser micrometer in *deep* and *normal* mode.

BPM	Stroke in mm *Deep* Mode	Stroke in mm *Normal* Mode
5	0.305	0.136
10	0.644	0.273
20	1.344	0.589
30	1.965	0.928
40	2.591	1.285
45	2.797	1.394
50	3.047	1.66
55	3.285	1.72
60	3.472	1.917
80	4.068	2.481

## Abbreviations

The following abbreviations are used in this manuscript:

ADC	analog-to-digital converter
BPM	breaths per minute
CW	Continuous Wave
FFT	Fast Fourier Transform
FMCW	Frequency Modulated Continuous Wave
FoV	field of view
GUI	Graphical user interface
IQ	in-phase and quadrature
ISM	Industrial, Scientific and Medical
IR-UWB	Impulse-Radio Ultra-Wideband
NICU	Neonatal Intensive Care Unit
PCL	Point Cloud Library
PSD	Power Spectral Density
RGB	Red Green Blue
RGB-D	Red, Green, Blue -Depth
ROS	Robot Operating System
SMIL	self- and mutually injection-locked
SNR	Signal-to-noise ratio
ToF	time-of-flight
UDP	User Datagram Protocol
UWB	Ultra-Wideband

## References

[1] Abbas A.K., Heimann K., Jergus K., Orlikowsky T., Leonhardt S. (2011). Neonatal non-contact respiratory monitoring based on real-time infrared thermography. BioMed. Eng. OnLine.

[2] Blanik N., Abbas A.K., Venema B., Blazek V., Leonhardt S. (2014). Hybrid optical imaging technology for long-term remote monitoring of skin perfusion and temperature behavior. J. Biomed. Opt..

[3] Pereira C.B., Heimann K., Venema B., Blazek V., Czaplik M., Leonhardt S. Estimation of respiratory rate from thermal videos of preterm infants. Proceedings of the Annual International Conference of the IEEE Engineering in Medicine and Biology Society, EMBS.

[4] Pereira C.B., Yu X., Member S., Goos T., Reiss I., Orlikowsky T., Heimann K., Venema B., Blazek V., Leonhardt S. (2019). Noncontact Monitoring of Respiratory Rate in Newborn Infants Using Thermal Imaging. IEEE Trans. Biomed. Eng..

[5] Klaessens J.H., van den Born M., van der Veen A., Sikkens-van de Kraats J., van den Dungen F.A., Verdaasdonk R.M. (2014). Development of a baby friendly non-contact method for measuring vital signs: First results of clinical measurements in an open incubator at a neonatal intensive care unit. Proc. SPIE.

[6] Jorge J., Villarroel M., Chaichulee S., Guazzi A., Davis S., Green G., McCormick K., Tarassenko L. Non-Contact Monitoring of Respiration in the Neonatal Intensive Care Unit. Proceedings of the 12th IEEE International Conference on Automatic Face and Gesture Recognition.

[7] Al-Naji A., Chahl J. (2016). Remote respiratory monitoring system based on developing motion magnification technique. Biomed. Signal Process. Control.

[8] Lorato I., Stuijk S., Meftah M., Verkruijsse W., De Haan G. Camera-based on-line short cessation of breathing detection. Proceedings of the 2019 International Conference on Computer Vision Workshop, ICCVW 2019.

[9] Wu H.Y., Rubinstein M., Shih E., Guttag J., Durand F., Freeman W. (2012). Eulerian video magnification for revealing subtle changes in the world. ACM Trans. Graph..

[10] Wijenayake U., Park S.Y. (2017). Real-time external respiratory motion measuring technique using an RGB-D camera and principal component analysis. Sensors.

[11] Procházka A., Schätz M., Vyšata O., Vališ M. (2016). Microsoft Kinect visual and depth sensors for breathing and heart rate analysis. Sensors.

[12] Bernacchia N., Scalise L., Casacanditella L., Ercoli I., Marchionni P., Tomasini E.P. Non contact measurement of heart and respiration rates based on Kinect™. Proceedings of the IEEE International Symposium on Medical Measurements and Applications MeMeA).

[13] Rihana S., Younes E., Visvikis D., Fayad H. Kinect2—Respiratory movement detection study. Proceedings of the 2016 38th Annual International Conference of the IEEE Engineering in Medicine and Biology Society (EMBC).

[14] Benetazzo F., Freddi A., Monteriù A., Longhi S. (2014). Respiratory rate detection algorithm based on RGB-D camera: Theoretical background and experimental results. Healthcare Technol. Lett..

[15] Lauhkonen E., Cooper B.G., Iles R. (2019). Mini review shows that structured light plethysmography provides a non-contact method for evaluating breathing patterns in children. Acta Paediat. Int. J. Paediat..

[16] Ghezzi M., Tenero L., Piazza M., Bodini A., Piacentini G. (2017). Structured Light Plethysmography (SLP): Management and follow up of a paediatric patient with pneumonia. Resp. Med. Case Rep..

[17] Hmeidi H., Motamedi-Fakhr S., Chadwick E.K., Gilchrist F.J., Lenney W., Iles R., Wilson R.C., Alexander J. (2018). Tidal breathing parameters measured by structured light plethysmography in children aged 2–12 years recovering from acute asthma/wheeze compared with healthy children. Physiol. Rep..

[18] Hesse N., Pujades S., Black M.J., Arens M., Hofmann U.G., Schroeder A.S. (2018). Learning and tracking the 3d body shape of freely moving infants from rgb-d sequences. arXiv.

[19] Giancola S., Valenti M., Sala R. (2018). State-of-the-Art Devices Comparison. A Survey on 3D Cameras: Metrological Comparison of Time-of-Flight, Structured-Light and Active Stereoscopy Technologies.

[20] Martinez M., Stiefelhagen R. Breathing rate monitoring during sleep from a depth camera under real-life conditions. Proceedings of the 2017 IEEE Winter Conference on Applications of Computer Vision, WACV 2017.

[21] Penne J., Schaller C., Hornegger J., Kuwert T. (2008). Robust real-time 3D respiratory motion detection using time-of-flight cameras. Int. J. Comput. Assisted Radiol. Surg..

[22] Coronel C., Wiesmeyr C., Garn H., Kohn B., Wimmer M., Mandl M., Glos M., Penzel T., Klosch G., Stefanic-Kejik A. (2020). 3D Camera and Pulse Oximeter for Respiratory Events Detection. IEEE J. Biomed. Health Inform..

[23] Rehouma H., Noumeir R., Jouvet P., Bouachir W., Essouri S. A computer vision method for respiratory monitoring in intensive care environment using RGB-D cameras. Proceedings of the 7th International Conference on Image Processing Theory, Tools and Applications, IPTA 2017.

[24] Rehouma H., Noumeir R., Bouachir W., Jouvet P., Essouri S. (2018). 3D imaging system for respiratory monitoring in pediatric intensive care environment. Comput. Med. Imag. Graph..

[25] Cenci A., Liciotti D., Frontoni E., Mancini A., Zingaretti P. Non-Contact monitoring of preterm infants using rgb-D camera. Proceedings of the ASME Design Engineering Technical Conference.

[26] Gleichauf J., Niebler C., Koelpin A. Automatic non-contact monitoring of the respiratory rate of neonates using a structured light camera. Proceedings of the 2020 42nd Annual International Conference of the IEEE Engineering in Medicine Biology Society (EMBC).

[27] Wolff C. radartutorial.eu. https://www.radartutorial.eu/.

[28] Kim J.D., Lee W.H., Lee Y., Lee H.J., Cha T., Kim S.H., Song K.M., Lim Y.H., Cho S.H., Cho S.H. (2019). Non-contact respiration monitoring using impulse radio ultrawideband radar in neonates. R. Soc. Open Sci..

[29] Schleicher B., Nasr I., Trasser A., Schumacher H. (2013). IR-UWB radar demonstrator for ultra-fine movement detection and vital-sign monitoring. IEEE Trans. Microw. Theor. Techniq..

[30] Tupin J.P. (2012). Ultra Wideband (UWB) Baby Monitors for Detection of Infant Cardiopulmonary Distress. U.S. Patent.

[31] Wang S., Pohl A., Jaeschke T., Czaplik M., Kony M., Leonhardt S., Pohl N. A novel ultra-wideband 80 GHz FMCW radar system for contactless monitoring of vital signs. Proceedings of the Annual International Conference of the IEEE Engineering in Medicine and Biology Society, EMBS.

[32] Matthews G., Sudduth B., Burrow M. (2000). A Non-Contact Vital Signs Monitor. Crit. Rev. Biomed. Eng..

[33] Marnach A., Schmiech D., DIewald A.R. Verification of algorithm for an I/Q-radar system for breathing detection in an incubator. Proceedings of the 2019 21st International Conference on Electromagnetics in Advanced Applications, ICEAA 2019.

[34] Schmiech D., Marnach A., Diewald A.R. (2019). Verification and first test measurement of a microwave-based vital sign monitor. Adv. Radio Sci..

[35] Li C., Cummings J., Lam J., Graves E., Wu W. (2009). Radar remote monitoring of vital signs. IEEE Microw. Mag..

[36] Yan Y., Li C., Yu X., Weiss M.D., Lin J. Verification of a non-contact vital sign monitoring system using an infant simulator. Proceedings of the 31st Annual International Conference of the IEEE Engineering in Medicine and Biology Society.

[37] Pisa S., Bernardi P., Cicchetti R., Giusto R., Pittella E., Piuzzi E., Testa O. (2014). Comparison between UWB and CW radar sensors for breath activity monitoring. Radar Sens. Technol..

[38] Linz S., Vinci G., Mann S., Lindner S., Barbon F., Weigel R., Koelpin A. (2013). A compact, versatile six-port radar module for industrial and medical applications. J. Electric. Comput. Eng..

[39] Koelpin A., Vinci G., Laemmle B., Kissinger D., Weigel R., Koelpin A., Vinci G., Laemmle B., Kissinger D., Weigel R. (2010). The Six-Port in Modern Society. IEEE Microw. Mag..

[40] Vinci G., Lenhard T., Will C., Koelpin A. Microwave interferometer radar-based vital sign detection for driver monitoring systems. Proceedings of the 2015 IEEE MTT-S International Conference on Microwaves for Intelligent Mobility, ICMIM 2015.

[41] Vinci G., Lindner S., Barbon F., Mann S., Hofmann M., Duda A., Weigel R., Koelpin A. (2013). Six-port radar sensor for remote respiration rate and heartbeat vital-sign monitoring. IEEE Trans. Microw. Theor. Techniq..

[42] Marchionni P., Scalise L., Ercoli I., Tomasini E.P. (2013). An optical measurement method for the simultaneous assessment of respiration and heart rates in preterm infants. Rev. Sci. Instrum..

[43] Nguyen P., Transue S., Choi M.H., Halbower A.C., Vu T. WiKiSpiro: Non-contact respiration volume monitoring during sleep. Proceedings of the Annual International Conference on Mobile Computing and Networking, MOBICOM.

[44] Will C., Shi K., Weigel R., Koelpin A. Advanced Template Matching Algorithm for Instantaneous Heartbeat Detection using Continuous Wave Radar Systems. Proceedings of the 2017 First IEEE MTT-S International Microwave Bio Conference (IMBIOC).

[45] Shi K., Will C., Steigleder T., Michler F., Weigel R., Ostgathe C., Koelpin A. A contactless system for continuous vital sign monitoring in palliative and intensive care. Proceedings of the 12th Annual IEEE International Systems Conference, SysCon 2018.

[46] Tang M., Kuo C., Wun D., Wang F., Horng T. (2016). A Self- and Mutually Injection-Locked Radar System for Monitoring Vital Signs in Real Time With Random Body Movement Cancellation. IEEE Trans. Microw. Theor. Techniq..

[47] SIMCharacters SIMCharacters High Emotion Simulation. https://www.simcharacters.com/jart/prj3/sim/main.jart?rel=de&content-id=1543323934281&reserve-mode=active.

[48] Laerdal Premature Anne. https://laerdal.com/de/products/simulation-training/obstetrics-paediatrics/premature-anne/.

[49] BabySIM User Guide. https://caehealthcare.com/media/files/User_Guides/BabySIM-User-Guide.pdf.

[50] XeThru Bot Simulates Breathing for Sensor Testing: Built from Lego. https://www.eenewseurope.com/news/xethru-bot-simulates-breathing-sensor-testing-built-lego.

[51] Innovativer Inkubator für Frühgeborene (SINOPE-NEO). https://www.gesundheitsforschung-bmbf.de/de/innovativer-inkubator-fur-fruhgeborene-sinope-neo-8623.php.

[52] pmd FAQ. https://pmdtec.com/picofamily/faq/.

[53] May S. (2009). 3D Time-of-Flight Ranging for Robotic Perception in Dynamic Environments. Ph.D. Thesis.

[54] Lange R. (2000). 3D Time-of-Flight Distance Measurement with Custom Solid-State Image Sensors in CMOS/CCD-Technology. Ph.D. Thesis.

[55] Rusu R.B., Marton Z.C., Blodow N., Dolha M., Beetz M. (2008). Towards 3D Point cloud based object maps for household environments. Robot. Autonom. Syst..

[56] Ramachandran G., Singh M. (1989). Three-dimensional reconstruction of cardiac displacement patterns on the chest wall during the P, QRS and T-segments of the ECG by laser speckle inteferometry. Med. Biol. Eng. Comput..

[57] Betts J., Desaix P., Johnson E., Johnson J., Korol O., Kruse D., Poe B., College O., Wise J., Womble M. (2013). Anatomy & Physiology.

[58] Reimann K. Interview: “Das Herz eines Neugeborenen ist nicht größer als eine Walnuss”. http://www.gesundheitsberater-berlin.de/praxis/krankheiten-von-a-z/kardiologie-fur-kinder/interview-das-herz-eines-neugeborenen-ist-nicht-grosser-als-eine-walnuss--2.

[59] Glossary, optoCONTROL. https://www.micro-epsilon.co.uk/service/glossar/optoCONTROL.html.

[60] Operating Instructions optoCONTROL 2520. https://www.micro-epsilon.co.uk/download/manuals/man--optoCONTROL-2520--en.pdf.

[61] Wiedemeyer T. PMD CamBoard Pico Flexx Driver. https://github.com/code-iai/pico_flexx_driver.

[62] CamBoard Pico Flexx. https://pmdtec.com/picofamily/wp-content/uploads/2018/03/PMD_DevKit_Brief_CB_pico_flexx_CE_V0218-1.pdf.

[63] GUARDIAN—Berührungslose Vitalparameterüberwachung Bietet mehr Lebensqualität für Pflegebedürftige Menschen. https://www.technik-zum-menschen-bringen.de/projekte/guardian.

[64] Conrad Components Stereo-Verstärker Bausatz 9 V/DC, 12 V/DC, 18 V/DC 35 W 2 Ohm. https://www.conrad.de/de/p/conrad-components-stereo-verstaerker-bausatz-9-v-dc-12-v-dc-18-v-dc-35-w-2-1216582.html.

[65] Visaton FR 10 4 Zoll 10.16 cm Breitband Lautsprecher-Chassis 20 W 4 Ohm. https://www.conrad.de/de/p/visaton-fr-10-4-zoll-10-16-cm-breitband-lautsprecher-chassis-20-w-4-303634.html.

[66] High Performance Laser Micrometer. https://www.micro-epsilon.co.uk/2D_3D/optical-micrometer/micrometer/optoCONTROL_2520/.

[67] Plane Model Segmentation. http://www.pointclouds.org/documentation/tutorials/planar_segmentation.html.

[68] Module Sample_Consensus. https://pointclouds.org/documentation/group__sample__consensus.html..

[69] PCL pcl::RadiusOutlierRemoval< pcl::PCLPointCloud2 >. https://pointclouds.org/documentation/classpcl_1_1_radius_outlier_removal_3_01pcl_1_1_p_c_l_point_cloud2_01_4.html.

[70] Ichim A.E. pcl::MedianFilter< PointT > Class Template Reference. https://pointclouds.org/documentation/classpcl_1_1_median_filter.html.

[71] Find-Peaks. https://github.com/claydergc/find-peaks.

[72] Gorry P.A. (1990). General least-squares smoothing and differentiation by the convolution (Savitzky-Golay) method. Anal. Chem..

[73] C++ Implementation of Savitzky-Golay filtering based on Gram polynomials. https://github.com/arntanguy/gram_savitzky_golay.

[74] FFTW. http://fftw.org/index.html.

[75] fit_ellipse. https://www.mathworks.com/matlabcentral/fileexchange/3215-fit_ellipse.

